# Identification and characterization of *TP53* gene Allele Dropout in Li-Fraumeni syndrome and Oral cancer cohorts

**DOI:** 10.1038/s41598-018-30238-7

**Published:** 2018-08-03

**Authors:** Mohammed Moquitul Haque, Pradnya Kowtal, Rajiv Sarin

**Affiliations:** 1 Sarin Lab, Advanced Centre for Treatment Research and Education in Cancer-Tata Memorial Centre, Kharghar, Navi Mumbai, Maharashtra India; 2 Homi Bhabha National Institute, Training School Complex, Anushakti Nagar, Mumbai, 400085 Maharashtra India; 3 Cancer Genetics Clinic, Tata Memorial Hospital, Tata Memorial Centre, Parel, Mumbai, 400012 Maharashtra India

## Abstract

Allele Drop out (ADO) arising from non-amplification of one allele may produce false negative result and impact clinical management. In cancer, germline and somatic genetic analysis is being increasingly used but the prevalence, nature and implications of ADO has not been studied in any cohort. In a cohort of 290 Li Fraumeni/Li Fraumeni Like Syndrome cases undergoing *TP53* genetic testing, of the 69 pathogenic mutations identified so far, 5 were initially missed and 4 were misgenotyped as homozygous mutation due to germline ADO. Of the 9 germline ADOs, 8 were sequence dependent, arising from a polymorphism (rs12951053) in the primer annealing region of exon 7. Of 35 somatic *TP53* variants identified by exome sequencing in 50 oral cancer tissues registered under International Cancer Genome Consortium (ICGC), as a result of ADO, 4 were not detectable and 6 were not called as variant on Sanger Sequencing due to low peak height. High prevalence of germline and somatic ADO in the most frequently mutated cancer gene *TP53*, highlights the need for systematic evaluation of ADO prevalence and causes in clinically important cancer genes. False negative result for high penetrance germline mutations or actionable somatic mutations in oncogenes could have major clinical implications.

## Introduction

Genotyping errors in germline or somatic mutation testing could have major clinical consequences for cancer patients and their families. Genotyping errors can occur due to a variety of factors including DNA sequence, sample quality, reagents, equipment and human factors^1^. In current clinical molecular diagnostics, the pre and post analytical errors have been greatly reduced with good laboratory practices and accreditation of laboratories. The analytical errors have also been reduced with careful design and validation of genotyping assays and external quality assurance (QA) programme^2^. However, Allele Dropout (ADO) remains an important analytical error in genotyping. ADO arises from insufficient amplification of one of the two alleles and the dropped allele remains below the detection threshold of sequencing. Dropout of the mutant allele causes false negative result while dropout of Wild Type (WT) allele makes a heterozygous mutation appear homozygous. The ADO is called sequence dependent when it occurs due to certain features within the sequence of the DNA being amplified. These include polymorphisms in the annealing region of the primers^3–5^, presence of tertiary structures like G-Quadruplexes and i-motifs^6,7^, methylation^6^ or allele size differences^8^. The sequence independent ADOs arise from poor DNA quality as in forensics^8^, Whole Genome Amplification (WGA) of scanty starting DNA as used in Single Cell Sequencing (SCS) or Preimplantation Genetic Diagnosis (PGD)^9^ and from unknown PCR conditions^8,10^.

ADOs as a cause of incorrect genotyping has been highlighted in diverse molecular diagnostic contexts^2,3,10–12^, but have not been systematically evaluated in oncology. It assumes greater importance in oncology as genetic analysis is being increasingly used for prognostication, precision medicine, hereditary risk assessment and cancer prevention. In the first systematic study of ADO in any cancer related gene, we have examined *TP53* gene (Mendelian Inheritance in Man-MIM*191170). This gene harbours a large number of well annotated germline and somatic mutations in cancer which are catalogued in International Agency for Research in Cancer (IARC) *TP53* database. *TP53* is the most frequently mutated gene in diverse cancer tissues^13^ and germline mutation in *TP53* is responsible for Li-Fraumeni Syndrome or Li-Fraumeni Like syndrome (LFS or LFL)^14^ - Mendelian Inheritance in Man (MIM) #151623.

## Results

### Germline Allele Dropout

#### Discovery Set

**Figure 1:**
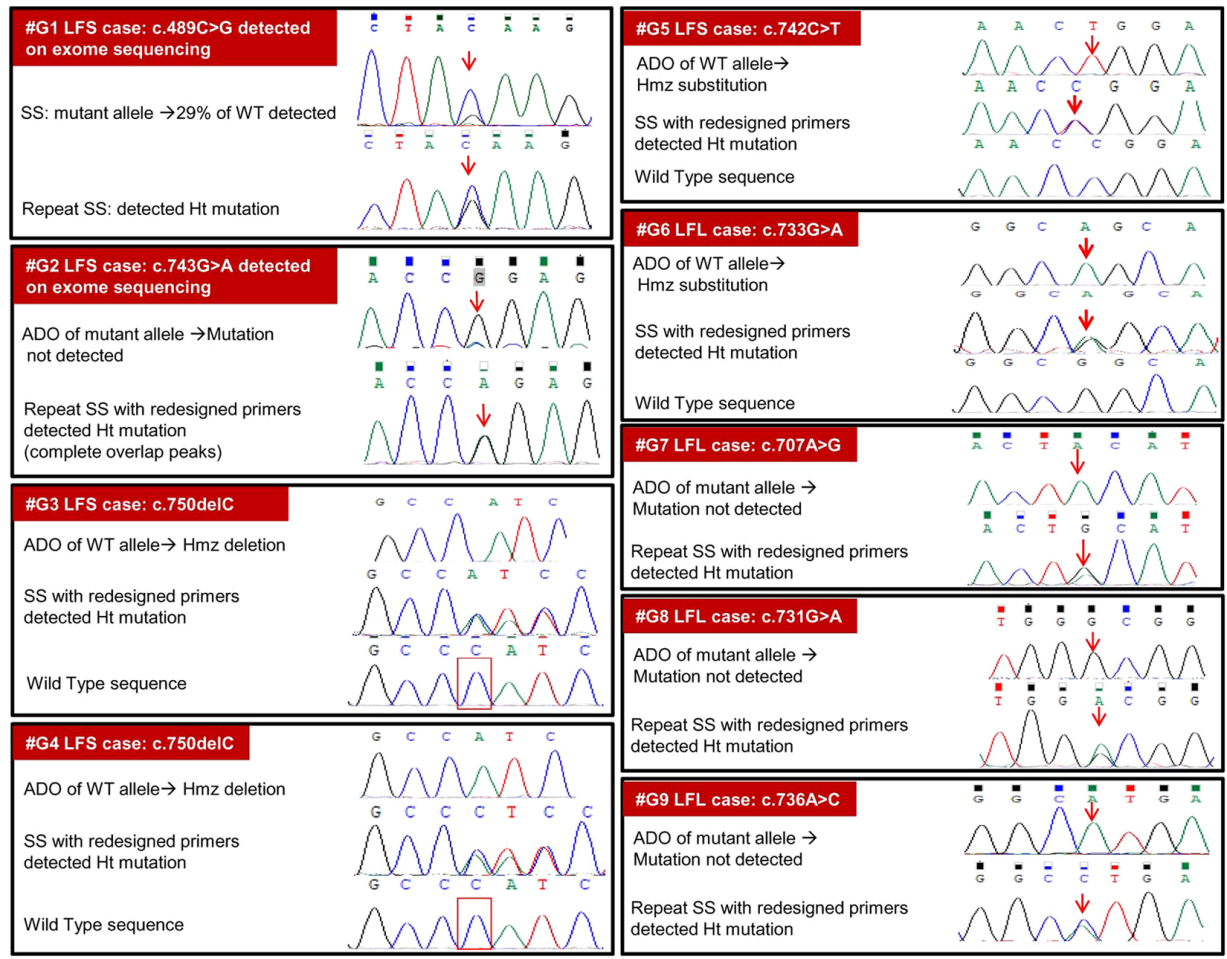
Germline *TP53* ADO in nine LFS/LFL cases: #G-Germline case number and syndromic diagnosis. #G1-6: ADO in discovery Set; #G7-9: ADO found in validation cohort; WT-Wild Type; Ht-Heterozygous; Hmz-Homozygous; LFS-Li Fraumeni Syndrome; LFL-Li Fraumeni like Syndrome; ADO-Allele Drop Out; SS-Sanger Sequencing. #G1- Exon 5 and #G2-G9- Exon 7.

Germline *TP53* mutations were tested in a cohort of 290 families. Of these, 150 families fulfilled the defined criteria of LFS or LFL^14^, while the remaining 140 families did not fulfil the criteria for LFS or LFL but were tested as either the proband or a family member had an LFS associated cancer. A total of 60 probands in this *TP53* tested cohort were found to carry a germline heterozygous mutation in *TP53*. In two classical LFS families germline whole exome sequencing was done as previous Sanger Sequencing had not identified any *TP53* mutation. In both these cases, a deleterious *TP53* germline mutation was identified on whole exome sequencing and later confirmed as ADO on repeat Sanger Sequencing (Fig. 1 #G1–2). One of the cases had mutation in exon 5, missed earlier due to low peak height and the other had a mutation in exon 7 that was detected by redesigned primers.

**Figure 2:**
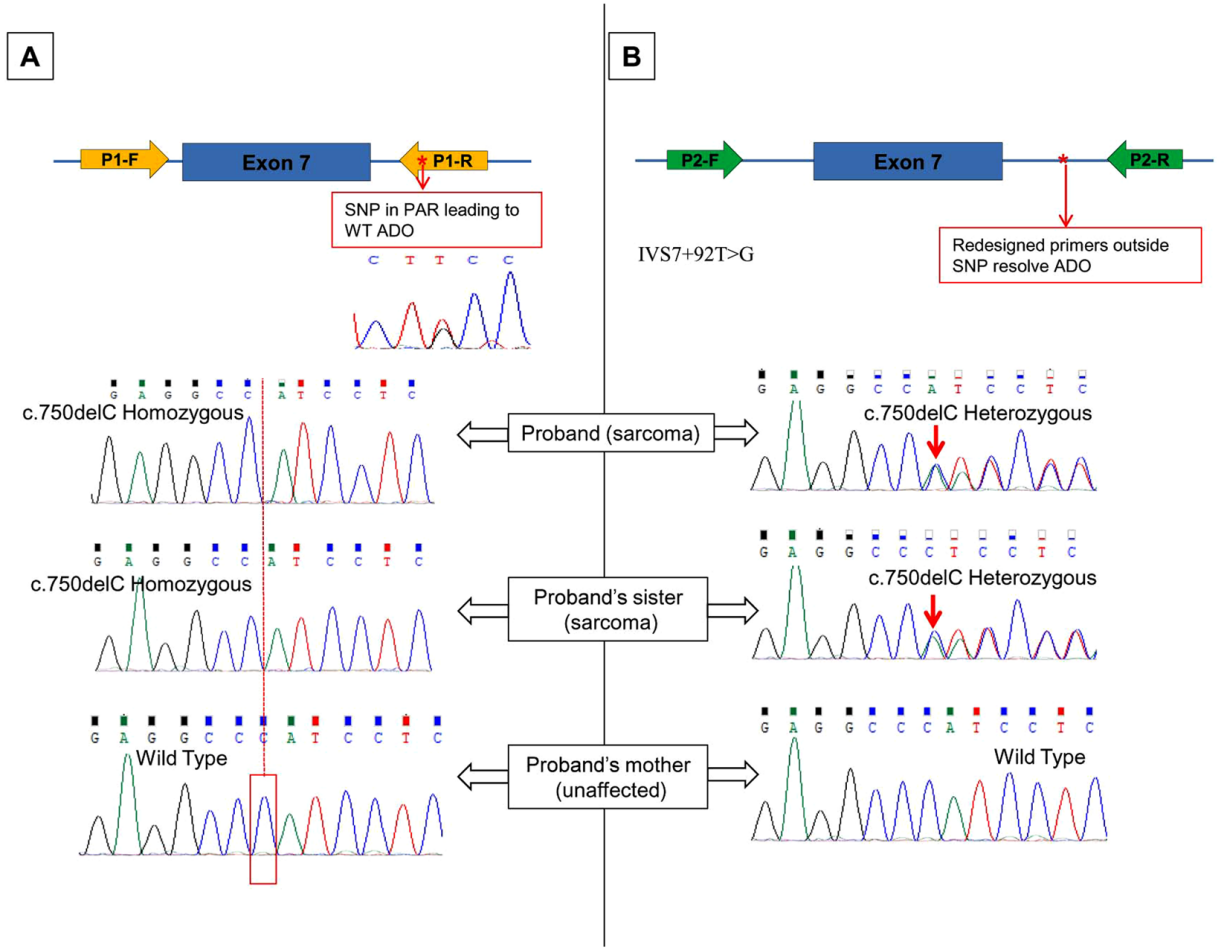
*TP53* Germline Allele dropout in an LFS family due to annealing region SNP: (**A**) Homozygous deletion in two sarcoma affected siblings. Mother unaffected and WT. Father who died of cancer was not tested. ADO of WT allele suspected and SNP (rs12951053) detected in the reverse primer (P1R) binding region. Chromatogram of the SNP is shown. The mutant allele is in the trans to the T allele of IVS7 + 92 T > G polymorphism and cis to the G allele.(**B**) Resequencing with redesigned primers (P2F and P2R) avoiding the SNP region confirmed ADO and detected heterozygous deletion mutation in the two siblings.

In four cases (#G3–6), ADO was suspected due to mutation homozygosity and confirmed on repeat sequencing with redesigned primers for exon 7 to avoid a common polymorphism as shown in Fig. 2. Therefore a total of 6 cases in the discovery set suspected of ADO were confirmed to have a heterozygous mutation on Sanger Sequencing.

#### Validation cohort

This consisted of 150 cases fulfilling the defined criteria of LFS or LFL in whom *TP53* full gene Sanger sequencing and MLPA had not identified any pathogenic mutation. In these 150 cases, chromatograms were read again to identify any possible variant that was not called earlier because the variant peak height ratio was <0.3 or it was >0.3 with background noise. In the first round of Sanger sequencing, 28 such suspected variants were identified. However on repeat Sanger sequencing using same conditions, none of these suspect variants could be detected and considered as artefacts (Fig. S1). For exon 7, sequencing was repeated with redesigned primers in these 150 cases and identified 3 additional germline ADOs (#G7-9). For exon 4–9 which has the DNA Binding Domain and harbours about 85% of all germline *TP53* mutations^15^, sequencing was repeated using same primers and conditions in 50 cases from the validation cohort. No additional ADO was identified in these 50 cases. A total of 9 germline ADO were detected, 6 from discovery set and 3 from validation set.

### Somatic Allele Dropout

**Figure 3:**
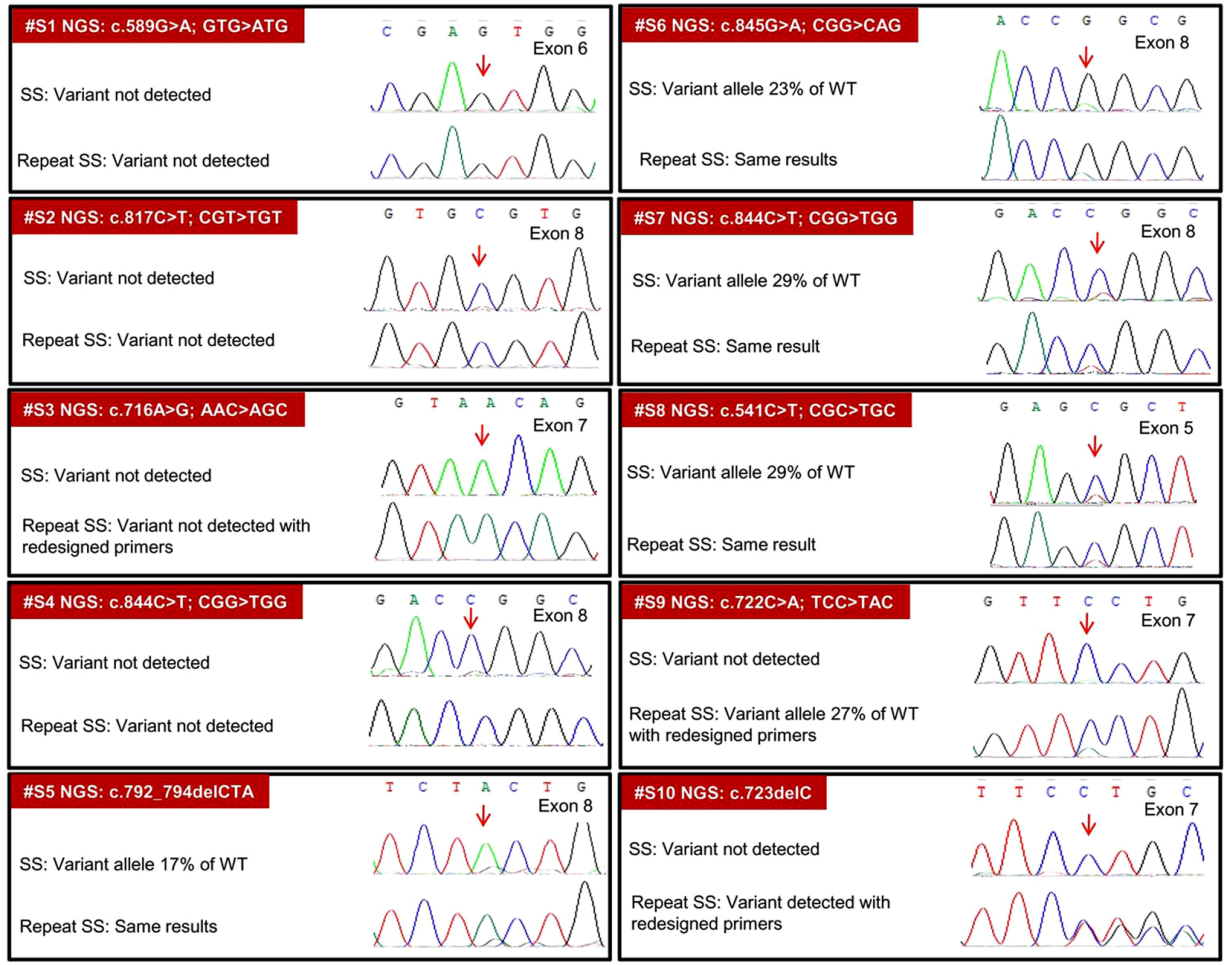
*TP53* Somatic ADO in Oral cancer tissue: Of the 35 *TP53* somatic variants in 50 tumors, 10 detected only by NGS but not by SS (ADO) were retested by SS.

Of the 35 *TP53* variants identified in 50 oral cancer tissues in the ICGC cohort^13^, somatic ADO was suspected on Sanger sequencing in 10 cases (Fig. 3). All these 10 somatic ADOs were earlier detected by orthogonal sequencing on two NGS platforms (Illumina HiSeq2000 and Roche GS-FLX) and further verified in Ion Torrent PGM (Life Technologies). Six exome variant were not visualized on Sanger sequencing (#S1-4, #S9-10) and 4 variants were visible but below the threshold peak ratio <0.3 (#S5-8). For these 10 suspected ADOs, Sanger sequencing was repeated with two different DNA concentrations - 100 ng per reaction as used initially and at an increased concentration of 200 ng. Same PCR conditions and primers were used, except for redesigned exon 7 primers. On repeat sequencing, 8 variants remained undetected (#S1-4) or below the 0.3 threshold as seen after initial sequencing (#S5-8). However, with redesigned exon 7 primers, one undetected variant was detected at peak ratio 0.27 (#S9) and another was clearly detected (#S10).

### Polymorphisms in *TP53* gene and PAR

In the dbSNP polymorphism database (NCBI), the *TP53* gene has 6148 polymorphisms in *Homo sapiens*. After specifying the variation class to SNP it showed the total number of SNP to be 5311. In the annealing region of the Bodmer group primer set that were used in our study, 58 polymorphic sites were identified with high minor allele frequency of >0.01 for one polymorphism in reverse primer of exon 7. In comparison, the annealing region of the most commonly used IARC protocol primer set (http://p53.iarc.fr/download/tp53_directsequencing_iarc.pdf, accessed on 12^th^ Mar 2018) harbours 83 polymorphisms and 4 of these have a minor allele frequency of >0.01 which affects five primers (Table 1).

**Table 1:** Comparison of polymorphisms in primer annealing region of Bodmer group Primers and IARC primers and their MAFs.

		Primers used in the study (Bodmer group primers)	IARC primers
		Number of Polymorphisms in PAR (MAF range)^	Number of polymorphisms in PAR (MAF range)^
1 F		1 (0.0007)	—
1 R		3 (0.00003)	—
2 F		2 (0.00003–0.0001)	—
2 R		7 (0.00003–0.0002)	—
2–3 F		—	2 (0.00002–0.00003)
2–3 R		—	7 (0.000008–0.0853*) rs17883323
3 + 4 F		3 (0.000008–0.0012)	—
3 + 4 R		2 (0.00003)	—
4 F		—	7 (0.000008–0.0113*) rs35117667
4 R		—	2 (0.000008–0.0002)
2^nd^ set	4 F	—	7 (0.000008-0.0853*) rs17883323
	4 R	—	1 (0.0001)
5 F		—	4 (0.000009-0.0002)
5 R		—	6 (0.000008-0.0002)
5 + 6 F		2 (0.0018–0.0073)	4 (0.00001–0.00003)
5 + 6 R		10 (0.00001–0.0034)	8 0.000009–0.0088)
6 F		—	3 (0.00002)
6 R		—	8 (0.000009–0.0088)
7 F		4 (0.0001–0.0002)	3 (0.000008–0.0002)
7 R		3 (0.0001–0.178*) rs12951053	2 (0.00003)
2^nd^ set	7 F	—	3 (0.00002–0.0127*) rs17880604
	7 R	—	5 (0.000008–0.0025)
8 F		—	4 (0.00002–0.0013)
8 R		—	3 (0.000008–0.0002)
8–9 F		2 (0.0002)	1 (0.0002)
8–9 R		2 (0.000008–0.00002)	3 (0.000008–0.0002)
9 F		—	5 (0.000008–0.0002)
9 R		—	3 (0.00002)
10 F		6 (0.00001–0.0002)	0
10 R		6 (0.00003–0.00007)	1 (0.0016)
11 F		1 (0.0002)	3 (0.00002–0.0112*) rs17881850
11 R		4 (0.00007–0.0001)	3 (0.0002–0.0014)
Total		58	98

Legend: ^Range of highest Minor Allele Frequency (MAF) as reported in different databases like 1000 Genomes database, TOPMED, ExAC, as documented in dbSNP of all the polymorphisms in the primer annealing region of *TP53*. *The frequency of polymorphisms higher than 1% and their rs id.

#### G-quadruplexes and methylation in *TP53* gene

The full *TP53* gene has 5931 regions including overlaps and 120 without overlaps G-rich sequences which can form quadruplex. The range of Quadruplex forming G-Rich Sequence (QGRS) score for these 5913 regions is 0–61. The coding DNA sequence of *TP53* gene has 192 regions including overlaps and 5 without overlaps having QGRS score ranging from 3–33. G-rich sequences in the different amplicons of *TP53* gene is mentioned in the Table S2. The maximum QGRS score for a 30 bp long sequence is 105^16^. No CpG islands were found in any amplicon of *TP53* gene except exon 1. Exon 1 being the promoter region has the CpG island.

## Discussion

The risk of wrong genotyping due to ADO exists in all amplification based genotyping methods. These include Fragment Length Polymorphism analysis, Sanger sequencing and NGS performed after target amplification^17^. In several fields such as forensics, population genetics, molecular ecology, single cell sequencing and prenatal genetic diagnosis, the possibility of misgenotyping due to ADO is always considered and corrected to the extent possible. In forensics and molecular ecology, the risk of ADO is due to the poor DNA quality or trace amounts of DNA with possible admixture of DNA from other individuals. In forensics there is a debate on the vagaries of Low Copy Number typing and its implications^18,19^. Ewens *et al*. have proposed three main strategies to identify genotyping errors - checking for Mendelian inheritance discrepancies, replicate analysis of samples and independent allele calling^20^. Using this strategy in the first systematic study of germline and somatic ADO in a cancer related gene, we report that ADOs resulted in missing ~7% pathogenic *TP53* germline mutations in Li Fraumeni syndrome and ~28% somatic *TP53* mutation in oral cancer tissues.

The higher somatic ADO rate as compared to the germline ADO rate with Sanger sequencing of the same gene using same primers and PCR conditions in our study may be due to several factors. These include replicative analysis by an orthogonal hybrid capture based NGS technique for somatic ADO versus same amplification based Sanger technique for germline ADO; higher frequency of somatic mutations in *TP53* as compared to germline mutations; and use of genetically heterogeneous tumour tissue admixed with normal tissue for somatic genotyping. A higher somatic ADO rate on genotyping using DNA of poor quality or extracted from scanty or heterogeneous tumour has been previously reported^21,22^. The minimum DNA and tissue requirement for molecular typing has been determined to be 1 ug DNA for which 9 cubic mm tissue or two 18 gauge needle cores of 1 cm length are required^22^. However in routine practice, inadequate quantity or quality of tumour tissue is common. In small tumour biopsies, multiple clones of cancer cells and a variable admixture with normal tissues could result in genotyping error. A recent French study in consecutive patients with metastatic lung adenocarcinoma found that 263/665 (39.5%) biopsies were of poor specimen adequacy due to low tumour cell percentage of <10% or absolute number of tumour cells being <100. In such samples, *KRAS* mutation detection rate by Real Time PCR SNaPshot assay was reduced to 15.8% as compared to 29.8% in FFPE specimens with >10% tumour cell or >100 tumour cells^19^. Such high rate of misgenotyping due to low copy number of the mutant allele is not unique to somatic genotyping. A recent study of re-analysis of blood DNA by NGS at very high depth of coverage above 500x in 108 patients with suspected LFS without detectable *TP53* mutations, identified 6 additional cases of mosaic germline *TP53* mutations^14^. Such high depth of coverage allowed heterozygous germline mutations to be confidently detected even when they were present in <5% reads.

In contrast to several systematic studies of somatic ADOs in oncogenes or tumour suppressor genes^11,21,23,24^, very scanty information exists regarding germline ADOs in genes responsible for hereditary cancers. Worldwide, a million or more cancer patients would have undergone germline genetic testing in the last two decades. However our systematic literature review could identify only 7 cases of germline ADO in any cancer predisposing gene^3,5,25,26^. This extreme rarity of reported germline ADOs in cancer genes raises an important warning that germline ADOs are usually not suspected, confirmed and reported. It is important to note that the seemingly high 7% false negative rate for germline *TP53* mutation in our study was established only through a systematic ADO evaluation in a large cohort of the monogenetic LFS/LFL syndrome. Moreover, it would have remained unnoticed without the serendipitous finding of homozygous deleterious *TP53* mutations in few families and Mendelian inheritance discrepancy in one family (Fig. 1). One particular polymorphism (IVS7 + 92 T > G) with high allele frequency of 0.16 in our cohort explains most of our germline ADOs as discussed later.

So far only two studies have systematically examined germline ADOs in molecular diagnostics^2,10^. In the multi-centre eMERGE-PGx study, genotyping errors for SNPs in 6 genes of pharmacogenomic relevance (*VKORC1, TMPT, SLCO1B1, DPYD, CYP2C9* and *CYP2C19*) were determined in 1792 cases. Each sample was genotyped independently in the participating research laboratories using NGS panel PGRNSeq at a mean depth of 496x and in clinical laboratories using orthogonal genotyping platforms like commercial ADME panels, Sanger sequencing or TaqMan or some other assays. All clinical laboratories were Clinical Laboratory Improvement Amendments (CLIA) approved. The overall genotyping discordance between research labs and the CLIA approved clinical labs was 2.8%. The research laboratories using NGS in Illumina Hiseq2000 or 2500 platform had no analytical errors or ADO but wrong genotyping occurred in 11/1792 (0.06%) samples due to pre-analytical errors like sample switching. In the CLIA approved clinical laboratories, wrong genotyping occurred in 26/1702 (1.5%) samples and 24 of these were due to ADO caused by polymorphism in the binding region of commercial genotyping assays^2^. Another large Canadian study of patients with various hereditary disorders studied the prevalence of germline ADOs in 30769 genotyping assays using Allele Specific Oligonucleotide (ASO) PCR for 8 specific mutations in *CFTR*, *CHE*, *FAH* and *SLC12A6* genes^10^. These ASO PCR assays were carefully designed and validated as per College of American Pathologist (CAP) guidelines. While no ADO was observed during external quality assurance (EQA) with CAP, allele dropout or dropin later occurred in 135/30769 (0.44%) genotype assays. Unlike the eMERGE-PGx study, 94% ADOs were due to sequence independent factors and only 6% were due to sequence dependent factors like polymorphism in the PAR.

In our LFS/LFL cohort, of the 69 pathogenic germline *TP53* mutations identified so far, 5 mutations were initially missed and 4 heterozygous mutations were incorrectly genotyped as homozygous due to ADOs. The redesigned exon 7 primers resolved 8/9 ADOs by avoiding the polymorphism IVS7 + 92 T > G which was found to have a high minor allele frequency of 0.16 (N = 112; TT = 80;TG = 28 & GG = 4) in our cohort and 0.17 in the 1000 genome database. The P1-7R primers we used, were initially described by Bodmer’s group^27,28^ and have been widely used, including the Children Oncology Group study correlating *TP53* mutations with sarcoma outcomes^29^, St Jude’s Children Hospital glioma study^30^ and several other studies^31–35^. The annealing region of the Bodmer group primer set harboured 58 polymorphic sites with high MAF for the IVS7 + 92 T > G polymorphism. The annealing regions of the widely used IARC protocol primers^36–39^ also has a larger number of polymorphisms and polymorphisms with MAF of >0.01(Tables 1and S3 and S4). Unfortunately the primer sequence is not described in majority of the recent publications and in commercial assays^2^. Hence it is difficult to estimate ADO probability in cohorts of individuals tested by different laboratories or institution and institute corrective measures.

A false negative test for a high penetrance germline mutation or an actionable oncogenic mutation could have major clinical implications^11^ as exemplified in our cohort. As a consequence of undetected germline *TP53* ADO, five of our families would not have been offered LFS screening for the probands and extended family testing. It is therefore imperative to minimize the possibility of ADO during the design, validation and quality assurance of the genotyping assays. ADOs originate during amplification process. NGS after amplification based target capture may therefore be as prone to sequence dependent ADOs as the Sanger sequencing^17^. Non-amplification methods like hybrid capture before NGS could minimize ADO probability and is useful for confirming sequence dependent ADO as in our study. Primer tiling with overlapping primers could minimize sequence dependent ADOs^17^. However, a recent report revealed that primer tiling without primer trimming had resulted in missing 2/174 germline *BRCA1/BRCA2* mutations^25^. Rarely, variants outside primer binding site can also cause ADO^40,41^. Bio-informatic flagging for homozygosity of rare variants is recommended to raise ADO alert^17^.

While the small gene size and minimal repetitive sequences in *TP53* would have a lower ADO risk, it may be more than offset by the large number of polymorphisms and an abundance of G-Quadruplexes (Table S2). Hence the prevalence and nature of ADOs in *TP53* may not be generalizable for other oncogenes or tumour suppressor genes. Nevertheless, our findings should prompt systematic large studies of ADO in diverse cancer cohorts, genotyped with different methods. This will help understand various clinical, genetic and technological contexts where ADOs could be a special consideration or require systematic corrective actions. ADO should be suspected for homozygous germline mutations in Mendelian Dominant conditions and in cases with classical syndromic diagnosis without a relevant gene mutation identified on amplification based genotyping and MLPA. Such cases or families, whether tested now or in the past, may be recalled for retesting by appropriate methods. Retrospective retesting of individuals with redesigned primers should be considered whenever any polymorphism with a significant allele frequency is identified in the annealing region of the primers used. The *TP53* exon 7 primers used in several studies including ours should never be used for somatic or germline *TP53* analysis.

In conclusion, germline and somatic ADO in *TP53* are not extremely rare and this may be true in other cancer genes. Considering the major clinical implications of ADOs, a systematic evaluation of ADOs in different clinical, genetic and technological contexts with appropriate remedial actions or retesting is warranted.

## Materials and Methods

### Patients

All patients in this ADO report were participants of studies approved by the Tata Memorial Centre-ACTREC Institutional Review Board. Written informed consent was obtained from all subjects for biobanking and genetic analysis. For minors, the written informed consent was provided by the parents. All experiments were carried out in accordance with the approved guidelines and regulations. Germline ADOs were examined in a cohort of cancer patients with personal or family history suggestive of hereditary LFS or LFL syndrome. These LFS/LFL families were enrolled in the Cancer Genetics Clinic of the Tata Memorial Hospital for genetic counseling and genetic testing. Germline *TP53* mutations were tested in a cohort of 290 families. Of these, 150 families fulfilled the defined criteria of LFS or LFL^14^, while the remaining 140 families did not fulfil the criteria for LFS or LFL but were tested as either the proband or a family member had an LFS associated cancer. Somatic ADOs were examined in a cohort of oral squamous carcinoma patients who had provided written informed consent for biobanking and germline and somatic genome analysis as part of the International Cancer Genome Consortium (ICGC) India project^13^.

#### Work Flow

In the germline ADO cohort of 290 LFS/LFL cases, the genomic DNA was extracted from peripheral blood lymphocytes by Qiagen columns (QIAamp DNA Blood Mini Kit; Cataloque number 51106) according to manufacturers protocol or in some cases by conventional phenol chloroform method. The entire coding region of the *TP53* gene was sequenced by Sanger sequencing. If no germline *TP53* mutation was identified, Large Genomic Rearrangement analysis was done by the Multiplex Ligation dependent Probe Amplification (MLPA) kit (MRC Holland) as per manufacturer’s instructions. In selected LFS/LFL cases without an identified *TP53* mutation on Sanger Sequencing and MLPA, either germline exome sequencing was done (n = 3) or targeted re-sequencing with multigene NGS panel in commercial laboratories (n = 8). In the Oral Cancer somatic ADO cohort, DNA was extracted from peripheral blood and tumour tissue with at least 60% viable tumour, using Qiagen columns (PAXgene Tissue DNA Kit (50). Exome sequencing was done on paired DNA from tumour and blood as described earlier^13^. In addition, Sanger sequencing for the entire *TP53* gene was done on tumour DNA for validation.

#### Polymerase Chain Reaction

For PCR amplification before Sanger Sequencing, we started with the commonly used primer set which were first described by the Bodmer group in 2006^27,28^. Exon 3 + 4 primer pair was redesigned in the initial phase due to poor amplification and exon 7 primer pair was redesigned when an ADO was suspected due to a common polymorphism in its annealing region. Primer sequences are described in Table S1. Annealing temperature for exon 1, 2, 5 + 6, 7 and 8 + 9 was 63.2 °C, for exon 10 and 11 was 57.8 °C and exon 3 + 4 was 58–51 °C touchdown PCR. The redesigned primers of amplicons 3 + 4 and 7 were amplified at annealing temperature of 68 °C and 66 °C respectively. PCR were set up in 25 ul volume with 10X PCR buffer (2.5 ul), 2.5 mM dNTPs (1 ul), 10 pmol primers (0.5 ul each), 20 ng/ul of gDNA (5 ul) and 1 unit of Finnzyme Taq polymerase (0.5 ul).

#### Sanger Sequencing

Amplified products were cleaned with Exonuclease and Shrimp Alkaline phosphatase for sequencing. 2 ul of cleaned PCR products with 1.5 pmol of primer is taken for cycle sequencing reaction. Post cycle sequencing products were sequenced with Big Dye Terminator kit version 2 (Applied Biosystems) on DNA sequencers 3500 Genetic Analyzer 8 capillary or 3730 DNA Analyzer 48 capillary (Applied Biosystems)

Chromatograms were analyzed using Chromas Lite and Sequencing Analysis Software v.5.3.1. The threshold for mixed base for detection of heterozygous mutations was set at a ratio of 0.3 of the wild type^23^, (https://tools.thermofisher.com/content/sfs/brochures/seq-quantification-app-note.pdf, accessed on 15^th^ Mar 2018)

#### Next Generation Sequencing (NGS)

Germline exome sequencing for LFS/LFL cases was carried out using Nextera rapid capture kit (Illumina) and sequenced on Hiseq2000 following manufacturer’s protocol. Commercial multi-gene panel which included a minimum of 25 genes recommended by American College of Medical Genetics and Genomics also used the Nextera rapid capture kit (Illumina) and sequencing is carried out using standard v2 kit on Illumina MiSeq. Exome for the oral cancer tissues to detect somatic mutations was captured using TruSeq exome enrichment kit (Illumina) and sequenced on Roche as reported previously^13^. The Nextera rapid capture kits is based on the principle of hybrid capture.

#### Multiplex Ligation Dependent Probe Amplification (MLPA)

MLPA was studied for large genomic rearrangements. MLPA was carried out according to manufacturer’s protocol (MRC Holland).

#### Polymorphisms within primers and G-quadruplexes

Total number of polymorphisms in *TP53* gene was searched in National Centre for Biotechnology Information (NCBI). SNP is selected from the dropdown menu and search word *TP53* is typed in the search box and further variation class was also specified (https://www.ncbi.nlm.nih.gov/snp, accessed on 5^th^ Apr 2018)

We searched the UCSC genome browser (https://genome.ucsc.edu/, accessed on 14^th^ Mar 2018) for polymorphisms within the primer annealing regions. For each polymorphism identified, its minor allele frequency (MAF) was searched in the dbSNP database by their rs ids (https://genome.ucsc.edu/, accessed on 14^th^ Mar 2018)

For the G-quadruplexes in the *TP53* sequence, the Quadruplex forming G-Rich Sequences (QGRS) Mapper was used^16^. The QGRS score for *TP53* gene is given in Table S2.

ADO was suspected if the *TP53* variant identified through exome sequencing was either not detected on Sanger sequencing or was detected with peak height ratio of <0.3 on the chromatogram. In addition, whenever a pathogenic germline mutation was detected in a homozygous state, we suspected a germline *TP53* ADO. Sanger sequencing was repeated for all suspected ADO samples in the discovery cohort and in a validation cohort of 150 LFS/LFL cases negative for *TP53* mutation on initial Sanger sequencing and MLPA. For exon 7 ADO, sequencing was repeated with redesigned primers as described in Fig. 1.

## Electronic supplementary material

Supplementary data
