# Supplementary material for: Identification and characterization of TP53 gene Allele Dropout in Li-Fraumeni syndrome and Oral cancer cohorts
Source: Sci Rep. 2018 Aug 3;8:11705. doi: 10.1038/s41598-018-30238-7 (PMC6076284; doi:10.1038/s41598-018-30238-7)
Supplement: Supplementary file 1 — Supplementary data [file 41598_2018_30238_MOESM1_ESM.docx]

**Supplementary Information**

**Identification and characterization of *TP53* gene Allele Dropout in Li-Fraumeni syndrome and Oral cancer cohorts.**

**Mohammed Moquitul Haque^1,2^, Pradnya Kowtal^1,2^, Rajiv Sarin^1,2,3^***

1. Sarin Lab, Advanced Centre for Treatment Research and Education in Cancer-Tata Memorial Centre, Kharghar, Navi Mumbai, Maharashtra, India.
2. Homi Bhabha National Institute, Training School Complex, Anushakti Nagar, Mumbai, 400085, Maharashtra, India.
3. Cancer Genetics Clinic, Tata Memorial Hospital, Tata Memorial Centre, Parel, Mumbai 400012, Maharashtra, India.

**Table S1: Primers and annealing temperatures**

| Amplicon | Primer | Annealing Temp |
| --- | --- | --- |
| Exon 1F | CACAGCTCTGGCTTGCAGA | 63.2°C |
| Exon 1R | AGCGATTTTCCCGAGCTGA |  |
| Exon 2F | AGCTGTCTCAGACACTGGCA | 63.2°C |
| Exon 2R | GAGCAGAAAGTCAGTCCCATG |  |
| Exon 3+4-P1-F | AGACCTATGGAAACTGTGAGTGGA | 58-51Touch Down |
| Exon 3+4-P1-R | GAAGCCTAAGGGTGAAGAGGA |  |
| Exon 3+4-P2-F^±^ | AGACCTATGGAAACTGTGAGTGGA | 68°C |
| Exon 3+4-P2-R^±^ | AGGAAGCCAAAGGGTGAAGAGG |  |
| Exon 5+6F | CGCTAGTGGGTTGCAGGA | 63.2°C |
| Exon 5+6R | CACTGACAACCACCCTTAAC |  |
| Exon 7-P1-F | CTGCTTGCCACAGGTCTC | 63.2°C |
| Exon 7-P1-R | TGGATGGGTAGTAGTATGGAAG |  |
| Exon 7-P2-F^±^ | AGAATGGCGTGAACCTGGGC | 66°C |
| Exon 7-P2-R^±^ | TCCATCTACTCCCAACCACC |  |
| Exon 8+9F | GTTGGGAGTAGATGGAGCCT | 63.2°C |
| Exon 8+9R | GGCATTTTGAGTGTTAGACTG |  |
| Exon 10F | CTCAGGTACTGTGTATATACTTAC | 57.8°C |
| Exon 10R | ATACACTGAGGCAAGAAT |  |
| Exon 11F | TCCCGTTGTCCCAGCCTT | 57.8°C |
| Exon 11R | TAACCCTTAACTGCAAGAACAT |  |

**Table S1**: Primer sequence and their annealing temperatures. ^15^ ^±^ Redesigned primers.

**Table S2: G-Quadruplex in *TP53* gene**

|  | | **Without overlaps** | **QGRS score range** | **Including overlaps** | **QGRS score range** |
| --- | --- | --- | --- | --- | --- |
| Full gene (19149 ntds) | | 120 | 5-61 | 5931 | 0-61 |
| CDS (1182 ntds) | | 5 | 3-33 | 192 | 3-33 |
| Amplicon | 1 (439ntds) | 2 | 18 | 75 | 2-18 |
|  | 2 (319ntds) | 3 | 16-21 | 122 | 1-21 |
|  | 3+4 (651ntds) | 4 | 8-40 | 2482 | 0-40 |
|  | 5+6 (550ntds) | 4 | 11-41 | 322 | 0-41 |
|  | 7 (283ntds) | 1 | 14 | 2 | 14 |
|  | 8+9 (455ntds) | 2 | 16 | 15 | 10-16 |
|  | 10 (348ntds) | 2 | 18-33 | 200 | 5-33 |
|  | 11 (476ntds) | 2 | 17-21 | 100 | 0-21 |

**Table S2:** **G-Quadruplex in *TP53* gene:** Calculated through Quadruplex forming G-Rich Sequence (QGRS) Mapper (<http://bioinformatics.ramapo.edu/QGRS/index.php>). Amplicons according to Bodmer group primers. CDS- Coding DNA Sequence, ntds- nucleotides.

**Table S3: Specific polymorphisms and their minor allele frequencies in the Bodmer group primers and redesigned primers**

| **Primer** | **rs-Id** | **Primer sequence (5’→3’)** | **Minor allele frequency** |
| --- | --- | --- | --- |
| 1F | rs542205900 | CACAGCTCTGGCTTGC*A*GA | -=0.0006/3 (1000 Genomes) -=0.0007/21 (TOPMED) |
| 1R | rs759135662 | AGCGATT*T*TCCCGAGCTGA | NA |
|  | rs950173336 | AGCGATTTTC*C*CGAGCTGA | NA |
|  | rs934950040 | AGCGATTTTCCCGA*G*CTGA | T=0.00003/1 (TOPMED) |
| 2F | rs946807916 | AGCTGTCTCAGA*C*ACTGGCA | A=0.00003/1 (TOPMED) |
|  | rs1044211651 | AGCTGTCT*C*AGACACTGGCA | T=0.0001/3 (TOPMED) |
| 2R | rs1051425969 | *G*AGCAGAAAGTCAGTCCCATG | NA |
|  | rs747908393 | GA*G*CAGAAAGTCAGTCCCATG | A=0.000008/1 (ExAC) A=0.00003/1 (TOPMED) |
|  | rs910296194 | GAGCA*G*AAAGTCAGTCCCATG | NA |
|  | rs769791585 | GAGCAGAAAGT*C*AGTCCCATG | G=0.000008/1 (ExAC) |
|  | rs943163238 | GAGCAGAAAGTCAGT*C*CCATG | NA |
|  | rs368444215 | GAGCAGAAAGTCAGTCCC*A*TG | G=0.00002/2 (ExAC) G=0.0002/2 (GO-ESP) G=0.00003/1 (TOPMED) |
|  | rs745951235 | GAGCAGAAAGTCAGTCCCAT*G* | A=0.00003/3 (ExAC) A=0.00003/1 (TOPMED) |
| 3+4F | rs748527030 | AGACCT*A*TGGAAACTGTGAGTGGA | C=0.000008/1 (ExAC) |
|  | rs1800369 | AGA*C*CTATGGAAACTGTGAGTGGA | A=0.0006/73 (ExAC) A=0.0012/6 (1000 Genomes) |
|  | rs786201754 | *A*GACCTATGGAAACTGTGAGTGGA | NA |
| 3+4R | rs1794286 | GAAGCC*T*AAGGGTGAAGAGGA | G=0.00003/1 (TOPMED) |
|  | rs888713050 | GAAGC*C*TAAGGGTGAAGAGGA | T=0.00003/1 (TOPMED) |
| 5+6F | rs145153611 | CGCTAGTGGGTTGCAGG*A* | A=0.0018/9 (1000 Genomes) A=0.0017/49 (TOPMED) |
|  | rs35850753 | C*G*CTAGTGGGTTGCAGGA | T=0.0056/28 (1000 Genomes) T=0.0073/213 (TOPMED) |
| 5+6R | rs766876306 | CA*C*TGACAACCACCCTTAAC | T=0.00001/1 (ExAC) |
|  | rs1014274675 | CACT*G*ACAACCACCCTTAAC | NA |
|  | rs763417136 | *C*ACTGACAACCACCCTTAAC | T=0.00001/1 (ExAC) |
|  | rs751098447 | CACTG*A*CAACCACCCTTAAC | G=0.00001/1 (ExAC) |
|  | rs17884607 | CACTGA*C*AACCACCCTTAAC | T=0.0019/179 (ExAC) T=0.0034/17 (1000 Genomes) T=0.00007/2 (TOPMED) |
|  | rs752280122 | CACTGACAACCAC*C*CTTAAC | A=0.00001/1 (ExAC) |
|  | rs755937328 | CACTGACAACCACCCT*T*AAC | A=0.00001/1 (ExAC) |
|  | rs777498958 | CACTGACAACCACCCTT*A*AC | T=0.00001/1 (ExAC) |
|  | rs749063728 | CACTGACAACCACCCTTA*A*C | G=0.00001/1 (ExAC) G=0.00003/1 (TOPMED) |
|  | rs587778001 | CACTGACAACCACCCTTAA*C* | A=0.00001/1 (ExAC) A=0.00003/1 (TOPMED) |
| 7F | rs559230724 | CTGCTTGCCAC*A*GGTCTC | C=0.000008/1 (ExAC) C=0.0002/1 (1000 Genomes) |
|  | rs866409111 | CTGCTTGCCA*C*AGGTCTC | NA |
|  | rs949871368 | CTGCTTGC*C*ACAGGTCTC | A=0.0001/3 (TOPMED) |
|  | rs530001801 | CTG*C*TTGCCACAGGTCTC | A=0.0002/1 (1000 Genomes) |
| 7R | rs753873488 | TGGATGG*GTA*GTAGTATGGAAG | NA |
|  | rs12951053  ¶ | TGGATGGGTAGTAGTATGG*A*AG | C=0.1783/893 (1000 Genomes) C=0.1012/2947 (TOPMED) |
|  | rs767106291 | TGGATGGGTAGTAGTATGGAA*G* | T=0.0001/3 (TOPMED) |
| 8+9F | rs189582361 | GTTGGGAGTAGATGGA*G*CCT | T=0.0002/1 (1000 Genomes) T=0.00003/1 (TOPMED) |
|  | rs546697065 | *G*TTGGGAGTAGATGGAGCCT | NA |
| 8+9R | rs749446092 | GGCATTTTG*A*GTGTTAGACTG | G=0.00002/2 (ExAC) |
|  | rs757347555 | GGCATTTTGAGTGTTAGAC*T*G | A=0.000008/1 (ExAC)) |
| 10F | rs774628036 | CTCAGGTACTGTGTATA*T*ACTTAC | G=0.00002/2 (ExAC) |
|  | rs2856754 | CTCAGGTACTGTG*T*ATATACTTAC | NA |
|  | rs759914394 | CTCAGGTACT*G*TGTATATACTTAC | T=0.000010/1 (ExAC) |
|  | rs555791463 | CTCAGGTA*C*TGTGTATATACTTAC | A=0.00003/3 (ExAC) A=0.0002/1 (1000 Genomes) |
|  | rs868745759 | CTCAGG*T*ACTGTGTATATACTTAC | NA |
|  | rs753236134 | C*T*CAGGTACTGTGTATATACTTAC | C=0.00001/1 (ExAC) C=0.00003/1 (TOPMED) |
| 10R | rs993124613 | *A*TACACTGAGGCAAGAAT | NA |
|  | rs951972108 | *AT*ACACTGAGGCAAGAAT | =0.00007/2 (TOPMED) |
|  | rs984707200 | ATA*C*ACTGAGGCAAGAAT | G=0.00007/2 (TOPMED) |
|  | rs750659817 | ATAC*A*CTGAGGCAAGAAT | NA |
|  | rs1026153616 | ATACAC*T*GAGGCAAGAAT | NA |
|  | rs910384618 | ATACACTGAGGCAAGAA*T* | C=0.00003/1 (TOPMED) |
| 11F | rs562130162 | TCCC*G*TTGTCCCAGCCTT | T=0.0002/1 (1000 Genomes) T=0.0001/3 (TOPMED) |
| 11R | rs771579509 | TAACCCTTAAC*T*GCAAGAACAT | NA |
|  | rs1027267843 | TAACCCTTAAC*T*GCAAGAACAT | C=0.00007/2 (TOPMED) |
|  | rs897923545 | TAACCCTTAACTG*C*AAGAACAT | NA |
|  | rs953090502 | TAACCCTTAACTGCAA*G*AACAT | A=0.0001/3 (TOPMED) |
| **Redesigned primers** | | | |
| 3+4-P2-F | rs1057517593 | AGACCTATGGAAACTGTGAGTGG*A* | NA |
|  | rs776933919 | AGACCTATGGAAACTGTGAGTG*G*A | T=0.000008/1 (ExAC) |
|  | rs748527030 | AGACCT*A*TGGAAACTGTGAGTGGA | C=0.000008/1 (ExAC) |
|  | rs1800369 | AGA*C*CTATGGAAACTGTGAGTGGA | A=0.0006/73 (ExAC) A=0.0012/6 (1000 Genomes) |
|  | rs786201754 | *A*GACCTATGGAAACTGTGAGTGGA | NA |
| 3+4-P2-R | rs888713050 | AGGAAGC*C*AAAGGGTGAAGAGG | T=0.00003/1 (TOPMED) |
|  | rs1794286 | AGGAAGCC*A*AAGGGTGAAGAGG | G=0.00003/1 (TOPMED) |
|  | rs781734539 | AGGAAGCCAAAGGGTGA*A*GAGG | G=0.0001/3 (TOPMED) |
| 7-P2-F | rs1008179602 | AGAATGGCGTGAACCTGGG*C* | C=0.00003/1 (TOPMED) |
|  | rs187553272 | AGAATGGC*G*TGAACCTGGG | T=0.0014/7 (1000 Genomes) T=0.0014/41 (TOPMED) |
| 7-P2-R | rs986535183 | TCCATCTACTCCCAACCAC*C* | T=0.00003/1 (TOPMED) |
|  | rs546697065 | TCCATCTACTCCCAA*C*CACC | NA |

**Table S3:** **Specific polymorphisms and their minor allele frequencies in the Bodmer group primers and redesigned primers**: Nucleotides in between two ‘*’ is the polymorphic site. Rows designated with “¶” indicates the MAF of polymorphism is >0.01 from any one of the database.

**Table S4: Specific polymorphisms and their minor allele frequencies in IARC protocol primers**

| **Primer** | **rs-Id** | **Primer sequence (5’→3’)** | **Minor allele frequency** |
| --- | --- | --- | --- |
| 2-3F | Rs759055064 | TCTCATGCTGGATCCC*C*ACT | T=0.00002/2 (ExAC) |
|  | rs922854842 | TCTCATG*C*TGGATCCCCACT | A=0.00003/1 (TOPMED) |
| 2-3R | rs757827281 | AGTCAGAGGACCAGGTCCT*C* | - =0.000008/1 (ExAC) |
|  | rs1052483026 | AGTCAGAGGACCA*G*GTCCTC | A=0.0001/3 (TOPMED) |
|  | rs754228616 | AGTCAGAGGA*C*CAGGTCCTC | T=0.000008/1 (ExAC) |
|  | rs764517800 | AGTCAGAGG*A*CCAGGTCCTC | G=0.000008/1 (ExAC) |
|  | rs17883323  ¶  (same polymorphism as in 2^nd^ 4F primer) | AGTCAGAGGACCAGGTC*C*TC | T=0.0659/7954 (ExAC) T=0.0777/389 (1000 Genomes) T=0.0708/921 (GO-ESP) T=0.0853/2483 (TOPMED) |
|  | rs200989844 | AGTCAG*A*GGACCAGGTCCTC | T=0.000008/1 (ExAC) T=0.0002/1 (1000 Genomes) |
|  | rs772244589 | AG*T*CAGAGGACCAGGTCCTC | C=0.000008/1 (ExAC) |
| 4F | rs786203749 | TGCTCTTTTCACCCATCTA*C* | NA |
|  | rs746791390 | TGCTCTTTTCACCCATCT*A*C | A=0.000008/1 (ExAC) |
|  | rs768373702 | TGCTCTTTTCACCCATC*T*AC | C=0.000008/1 (ExAC) |
|  | rs35117667  ¶ | TGCTCTTTTCACCCAT*C*TAC | A=0.0025/303 (ExAC) A=0.0076/38 (1000 Genomes) A=0.0092/120 (GO-ESP) A=0.0113/330 (TOPMED) |
|  | rs202217267 | TGCTCTTTTCACC*C*ATCTAC | A=0.0001/16 (ExAC) A=0.0002/2 (GO-ESP) A=0.00007/2 (TOPMED) |
|  | rs769697802 | TGCTCTTTTCA*C*CCATCTAC | A=0.00002/2 (ExAC) |
|  | rs374547451 | TGCTC*T*TTTCACCCATCTAC | G=0.000008/1 (ExAC) G=0.00008/1 (GO-ESP) |
| 4R | rs530718177 | ATACGGCCAGG*C*ATTGAAGT | T=0.0001/16 (ExAC) T=0.0002/1 (1000 Genomes) |
|  | rs759403255 | ATA*C*GGCCAGGCATTGAAGT | A=0.000008/1 (ExAC) |
| 2^nd^ 4F | rs772244589 | TGAGGACCTGGTCCTCTG*A*C | C=0.000008/1 (ExAC) |
|  | rs200989844 | TGAGGACCTGGTCC*T*CTGAC | T=0.000008/1 (ExAC) T=0.0002/1 (1000 Genomes) |
|  | rs17883323  ¶  Same as in 2-3R | TGAGGACCTGGTC*C*TCTGAC | T=0.0659/7954 (ExAC) T=0.0777/389 (1000 Genomes) T=0.0708/921 (GO-ESP) T=0.0853/2483 (TOPMED) |
|  | rs764517800 | TGAGGACCTGG*T*CCTCTGAC | G=0.000008/1 (ExAC) |
|  | rs754228616 | TGAGGACCTG*G*TCCTCTGAC | T=0.000008/1 (ExAC) |
|  | rs1052483026 | TGAGGAC*C*TGGTCCTCTGAC | A=0.0001/3 (TOPMED) |
|  | rs757827281 | *TG*AGGACCTGGTCCTCTGAC | -=0.000008/1 (ExAC) |
| 2^nd^ 4R | rs781734539 | *A*GAGGAATCCCAAAGTTCCA | G=0.0001/3 (TOPMED) |
| 5F | rs747705704 | TTCAACTCTGTCTCCTTCC*T* | C=0.000009/1 (ExAC) |
|  | rs376713749 | TTCAACTCTGTCT*C*CTTCCT | A=0.00004/4 (ExAC) A=0.0002/2 (GO-ESP) |
|  | rs756417643 | TTCAACTCTGTC*T*CCTTCCT | A=0.000009/1 (ExAC) |
|  | rs773029752 | TTCAACT*C*TGTCTCCTTCCT | A=0.00002/2 (ExAC) |
| 5R | rs748298015 | CA*G*CCCTGTCGTCTCTCCAG | A=0.00002/2 (ExAC) T=0.00003/1 (TOPMED) |
|  | rs778145407 | CAGCCCT*G*TCGTCTCTCCAG | C=0.00002/2 (ExAC) |
|  | rs56181208 | CAGCCCTGTC*G*TCTCTCCAG | A=0.0002/26 (ExAC) A=0.00008/1 (GO-ESP) A=0.00007/2 (TOPMED) |
|  | rs774831915 | CAGCCCTGTCG*T*CTCTCCAG | C=0.000008/1 (ExAC) |
|  | rs547244762 | CAGCCCTGTCGTCT*C*TCCAG | T=0.00006/7 (ExAC) T=0.0002/1 (1000 Genomes) |
|  | rs772637453 | CAGCCCTGTCGTCTCT*C*CAG | G=0.000008/1 (ExAC) |
| 6F | rs76962868 | GCCTCTGATTCCTCAC*T*GAT | NA |
|  | rs869158731 | GCCTCTGAT*T*CCTCACTGAT | NA |
|  | rs757029874 | GCCTCT*G*ATTCCTCACTGAT | T=0.00002/2 (ExAC) |
| 6R | rs755937328 | T*T*AACCCCTCCTCCCAGAGA | A=0.00001/1 (ExAC) |
|  | rs777498958 | TT*A*ACCCCTCCTCCCAGAGA | T=0.00001/1 (ExAC) |
|  | rs749063728 | TTA*A*CCCCTCCTCCCAGAGA | G=0.00001/1 (ExAC) G=0.00003/1 (TOPMED) |
|  | rs587778001 | TTAA*C*CCCTCCTCCCAGAGA | A=0.00001/1 (ExAC) A=0.00003/1 (TOPMED) |
|  | rs778894743 | TTAAC*C*CCTCCTCCCAGAGA | G=0.00002/2 (ExAC) |
|  | rs34949160 | TTAACCCC*T*CCTCCCAGAGA | C=0.0088/880 (ExAC) C=0.0050/25 (1000 Genomes) C=0.0037/48 (GO-ESP) C=0.0041/118 (TOPMED) |
|  | rs200372146 | TTAACCCCT*C*CTCCCAGAGA | T=0.0002/1 (1000 Genomes) T=0.0002/5 (TOPMED) |
|  | rs762077893 | TTAACCCCTCCTCCCAGA*G*A | C=0.000009/1 (ExAC) |
| 5-6F | rs576912263 | TGTTCACTTGTGCCC*T*GACT | G=0.00001/1 (ExAC) |
|  | rs770686190 | TGTTCACTTG*T*GCCCTGACT | G=0.00001/1 (ExAC) |
|  | rs994417264 | TGTTCA*C*TTGTGCCCTGACT | A=0.00003/1 (TOPMED) |
|  | rs774330271 | TGT*T*CACTTGTGCCCTGACT | C=0.00001/1 (ExAC) |
| 5-6R | (same as 6R) | TTAACCCCTCCTCCCAGAGA |  |
| 7F | rs559230724 | CTTGCCAC*A*GGTCTCCCCAA | C=0.000008/1 (ExAC) C=0.0002/1 (1000 Genomes) |
|  | rs866409111 | CTTGCCA*C*AGGTCTCCCCAA | NA |
|  | rs530001801 | *C*TTGCCACAGGTCTCCCCAA | A=0.0002/1 (1000 Genomes) |
| 7R | rs1642786 | AGGGGTCAG*A*GGCAAGCAGA | NA |
|  | rs1036988722 | AGGGGTCAGA*G*GCAAGCAGA | A=0.00003/1 (TOPMED) |
| 2^nd^ 7F | rs769521263 | AGGCGCACTG*G*CCTCATCTT | T=0.00003/3 (ExAC) |
|  | rs17880604  ¶ | AGGC*G*CACTGGCCTCATCTT | G=0.0127/1527 (ExAC) G=0.0050/25 (1000 Genomes) G=0.0115/150 (GO-ESP) G=0.0102/296 (TOPMED) |
|  | rs374907737 | AGG*C*GCACTGGCCTCATCTT | A=0.0003/31 (ExAC) A=0.0002/1 (1000 Genomes) A=0.0004/5 (GO-ESP) |
| 2^nd^ 7R | rs756510290 | *T*GTGCAGGGTGGCAAGTGGC | C=0.000008/1 (ExAC) |
|  | rs201930255 | TGTGCA*G*GGTGGCAAGTGGC | T=0.00006/7 (ExAC) C=0.0002/1 (1000 Genomes) T=0.0002/2 (GO-ESP) T=0.0001/4 (TOPMED) |
|  | rs17880172 | TGTGCAGGGT*G*GCAAGTGGC | A=0.0006/71 (ExAC) A=0.0014/7 (1000 Genomes) A=0.0025/33 (GO-ESP) A=0.0018/52 (TOPMED) |
|  | rs17881780 | TGTGCAGGGTGGCAA*G*TGGC | A=0.00007/8 (ExAC) A=0.00008/1 (GO-ESP) A=0.0002/6 (TOPMED) |
|  | rs200277687 | TGTGCAGGGTGGCAAGT*G*GC | A=0.00002/2 (ExAC) A=0.0002/1 (1000 Genomes) |
| 8F | rs113302588 | TTCCTTAC*T*GCCTCTTGCTT | G=0.0002/14 (ExAC) G=0.0003/4 (GO-ESP) G=0.0013/37 (TOPMED) |
|  | rs376988747 | TTCCTTA*C*TGCCTCTTGCTT | A=0.00002/1 (ExAC) A=0.00008/1 (GO-ESP) A=0.00007/2 (TOPMED) |
|  | rs776659879 | TTCCTT*A*CTGCCTCTTGCTT | C=0.00007/4 (ExAC) C=0.0001/4 (TOPMED) |
|  | rs761687865 | TTCCT*T*ACTGCCTCTTGCTT | NA |
| 8R | rs144496254 | AGGCATAAC*T*GCACCCTTGG | A=0.00002/2 (ExAC) C=0.0002/1 (1000 Genomes) |
|  | rs945036538 | AGGCATAACT*G*CACCCTTGG | A=0.00007/2 (TOPMED) |
|  | rs747431888 | AGGCATAACTGCACC*C*TTGG | G=0.000008/1 (ExAC) |
| 8-9F | rs189582361 | TTGGGAGTAGATGGA*G*CCT | T=0.0002/1 (1000 Genomes) T=0.00003/1 (TOPMED) |
| 8-9R | rs749446092 | *A*GTGTTAGACTGGAAACTTT | G=0.00002/2 (ExAC) |
|  | rs757347555 | AGTGTTAGAC*T*GGAAACTTT | A=0.000008/1 (ExAC) |
|  | rs149983651 | AGTGTTAGACTGGAAA*C*TTT | T=0.00002/3 (ExAC) G=0.0002/1 (1000 Genomes) |
| 9F | rs768950740 | GACAAGAAGCGGT*G*GAG | T=0.000008/1 (ExAC) |
|  | rs376079415 | GACAAGAAGC*G*GTGGAG | T=0.00007/8 (ExAC) T=0.00008/1 (GO-ESP) T=0.0002/6 (TOPMED) |
|  | rs549731874 | GACAAGAAG*C*GGTGGAG | A=0.000008/1 (ExAC) A=0.0002/1 (1000 Genomes) A=0.00003/1 (TOPMED) |
|  | rs936119541 | *G*ACAAGAAGCGGTGGAG | -=0.00003/1 (TOPMED) |
|  | rs769211481 | *G*ACAAGAAGCGGTGGAG | A=0.000008/1 (ExAC) |
| 9R | rs769817504 | *C*GGCATTTTGAGTGTTAGAC | NA |
|  | rs567351657 | CGGC*A*TTTTGAGTGTTAGAC | NA |
|  | rs749446092 | CGGCATTTTG*A*GTGTTAGAC  (same polymorphism as in 8-9R) | G=0.00002/2 (ExAC) |
| 10F | No polymorphism | CAATTGTAACTTGAACCATC |  |
| 10R | rs375905156 | GGATGAGAATGGAATCCT*A*T | G=0.0008/4 (1000 Genomes) G=0.0016/46 (TOPMED) |
| 11F | rs750853458 | AGACCCTCTCA*C*TCATGTGA | A=0.00002/2 (ExAC) |
|  | rs17881850  ¶ | AGACC*C*TCTCACTCATGTGA | A=0.0112/1359 (ExAC) A=0.0076/38 (1000 Genomes) A=0.0088/115 (GO-ESP) A=0.0064/185 (TOPMED) |
|  | rs767047551 | AGA*C*CCTCTCACTCATGTGA | A=0.00002/3 (ExAC) |
| 11R | rs375913211 | TGA*C*GCACACCTATTGCAAG | T=0.0002/6 (TOPMED) |
|  | rs35919705 | TGAC*G*CACACCTATTGCAAG | A=0.0014/7 (1000 Genomes) A=0.0014/40 (TOPMED) |
|  | rs886053513 | TGACGCACAC*C*TATTGCAAG | NA |

**Table S4:** **Specific polymorphisms and their minor allele frequencies in IARC protocol primers**: (<http://p53.iarc.fr/download/tp53_directsequencing_iarc.pdf>): Nucleotides in between two ‘*’ is the polymorphic site. Rows designated with “¶” indicates the MAF of polymorphism is >0.01 from any one of the database.

.

F**igure S1**: 28 suspected variants in which Sanger sequencing was repeated


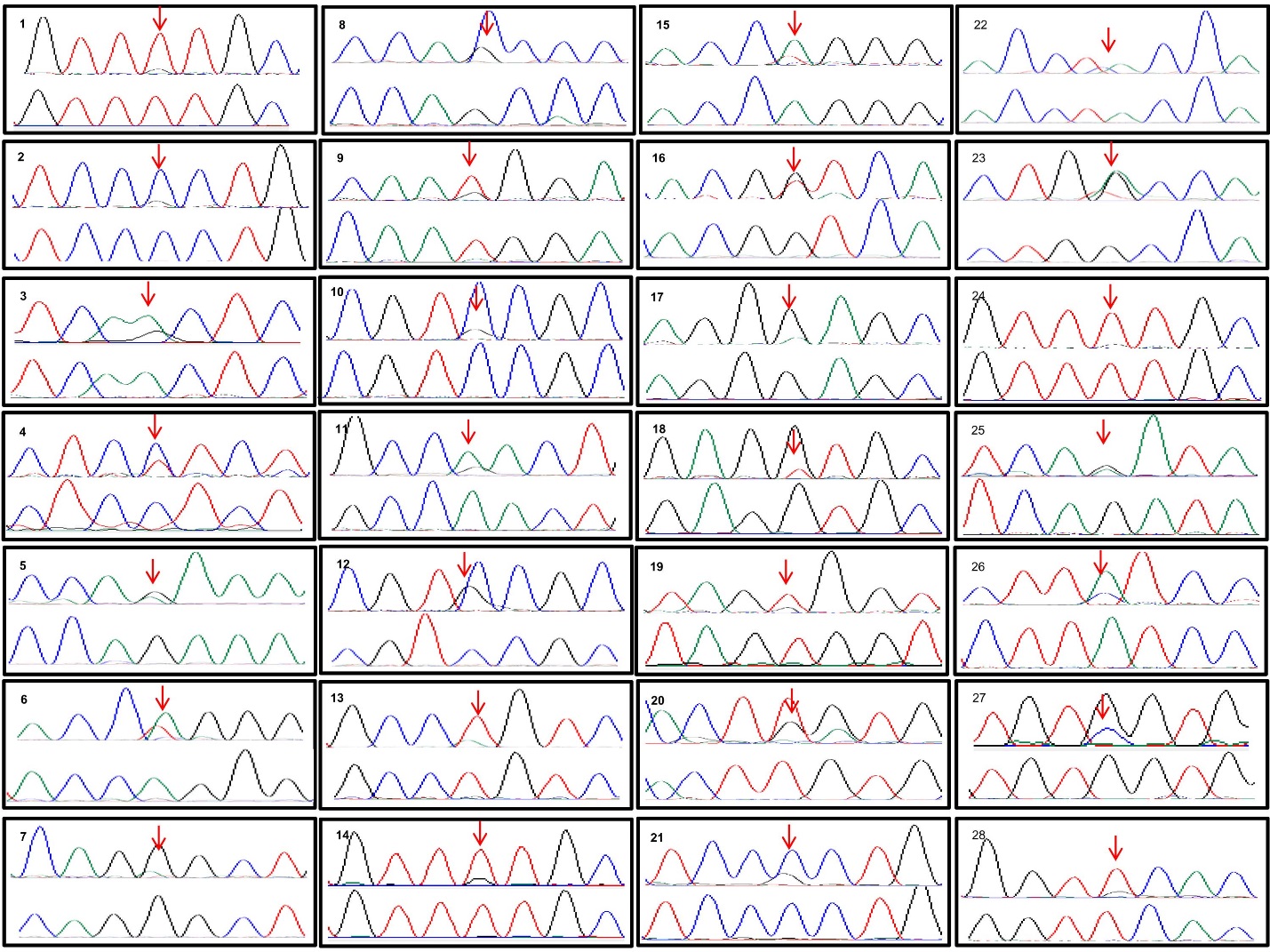


**Figure S1**: 28 suspected variants in Sanger Sequencing in which repeat Sanger sequencing was done using same conditions. None of these suspected variants could be detected and considered as artefacts.
